# 4579 Distinct clinical and immunological responses to αPD-1, kαPD-L1 and αPD-L2 immunotherapy in B16 melanoma in aged versus young hosts includes T-cell stem cell effects and PD-L2 expression differences

**DOI:** 10.1017/cts.2020.63

**Published:** 2020-07-29

**Authors:** Myrna G Garcia, Alvaro Padron, Yilun Deng, Harshita Gupta, Aravind Kancharla, Ryan Reyes, Tyler Curiel

**Affiliations:** 1 University of Texas Health Science Center San Antonio

## Abstract

OBJECTIVES/GOALS: Aging is the biggest risk factor for cancer, yet little is known about cancer immunotherapy effects. Here we investigate melanoma response to αPD-1, αPD-L1 and αPD-L2 in young vs. aged hosts. We look at different immune outcomes as possible mechanism. METHODS/STUDY POPULATION: We tested αPD-1 (100 μg/mouse), αPD-L1 (100 μg/mouse) or αPD-L2 (200 μg/mouse) in aged (18-24 months) and young (3-8 months) mice challenged orthotopically with B16. Tumors and draining lymph nodes (TDLN) were analyzed by flow. Bone marrow-derived DC were generated with GM-CSF. RESULTS/ANTICIPATED RESULTS: We reported that αPD-1 treats young and aged with B16 and αPD-L1 only treats young. aPD-L2 treated B16 in aged but, remarkably, not young, the first anti-cancer single agent immunotherapy exhibiting this property. Efficacy in young (aPD-1, aPD-L1) and aged (aPD-1, aPD-L2) correlated with increased T cell stem cells (TCSC) and total tumor-infiltrating lymphocytes (TIL), but TCSC differed by age and treatment (*e.g.*, distinct CCR2, CXCR5, CXCR3, PD-1 and TIM- expression). Aged expressed significantly more T-cell PD-1 and up to 40-fold more PD-L2 versus young in myeloid and NK cells, and TCSC. Bone marrow-derived DC experiments suggest aged DC are destined for high PD-L2 versus young. DISCUSSION/SIGNIFICANCE OF IMPACT: Treatment differences in aged vs. young could depend on immune checkpoint or TCSC differences. We are now identifying mechanisms for increased PD-L2 and contributions to aPD-L2 efficacy in aged, and testing TCSC effects. Our work can improve cancer immunotherapy in aged hosts and further provide important insights even in young hosts.

